# The National Dental Association

**Published:** 1898-07

**Authors:** 


### The National Dental Association.

At the last meeting of the National Dental Association, the following was adopted :

"*Resolved,* That a committee of three be appointed whose duty it shall be to confer with State local societies, with a view to aid and foster new societies when and where desirable."

The following were appointed the committee to act under these resolutions: J. Taft, Cincinnati; J. Y. Crawford, Nashville, Tenn.; James McManus, Hartford, Conn.

In obedience to these resolutions it is the desire of the committee to confer so far as possible with all State societies of the country and to obtain certain information in regard to the condition and work of each, in order that a report may be made at coming meeting of the association.

Time is brief for this work, but by prompt action on the part of the officers of the various societies to whom these notices may be sent, it is hoped that an interesting report may be made, and one that shall, in some degree at least, promote the interest of the National body.

